# How can fertility counseling be implemented for every newly diagnosed pediatric patient facing gonadotoxic treatment?-A single-center experience

**DOI:** 10.1007/s00277-021-04648-z

**Published:** 2021-09-18

**Authors:** Anke Barnbrock, Emilia Salzmann-Manrique, Nicole Sänger, Henning Fiegel, Falk Ochsendorf, Thomas Klingebiel, Peter Bader, Andrea Jarisch

**Affiliations:** 1 Division for Stem Cell Transplantation and Immunology, Department for Children and Adolescents, University Hospital, Goethe University, Frankfurt/Main, Germany; 2 Division for Pediatric Hematology and Oncology, Department for Children and Adolescents, University Hospital, Goethe University, Theodor-Stern-Kai 7, 60596 Frankfurt/Main, Germany; 3 Division for Gynecological Endocrinology and Reproductive Medicine, Department for Gynecology and Obstetrics, University Hospital Bonn, Bonn, Germany; 4 Division for Pediatric Surgery and Pediatric Urology, Department for Surgery, University Hospital, Goethe University, Frankfurt/Main, Germany; 5 Division for Andrology, Department for Dermatology, Venerology and Allergology, University Hospital, Goethe University, Frankfurt/Main, Germany

**Keywords:** Fertility counseling, Pediatric oncology, Pediatric stem cell transplantation, Indication for fertility preservation

## Abstract

Since the survival rates of pediatric patients undergoing cancer treatment or hematopoietic stem cell transplantation (HSCT) have increased rapidly in recent decades, the late effects of treatment are now an important focus of patient care. Access to fertility preservation (FP) procedures as well as their financing differs considerably across Europe. However, some countries in Europe have recently changed the legal basis for financing FP procedures; therefore, the implementation of structures is mandatory to give patients access to FP. In this prospective cohort study, we characterized the process for establishing pediatric fertility counseling, including the development of an in-house standard procedure for recommendations regarding FP with potentially gonadotoxic treatment and valuating data from all FP counseling sessions. All data concerning patient characteristics (pubertal status, disease group) and recommendation of FP measures were prospectively collected and adoption of FP measures analyzed. Prior to the establishment of a structured process for FP in our pediatric oncology and stem cell transplantation center, there was no standardized FP counseling. We demonstrate that with the establishment of an inhouse standard procedure, it is possible to give consistent yet individualized FP counseling to approximately 90% of our patients facing gonadotoxic treatment, counseling over 200 patients between 2017 and 2019. This pilot study could potentially be adapted in other pediatric hematology, oncology, and stem cell transplantation centers to allow a more standardized handling of FP counseling for all patients facing gonadotoxic treatment.

**Supplementary Information:**

The online version contains supplementary material available at 10.1007/s00277-021-04648-z.

## Introduction

With the immense progress in therapeutic regimens in pediatric oncology, the survival rates have increased up to 80% [1]. Therefore, broader indications for various diseases in the malignant and especially the nonmalignant settings have led to an increasing number of patients treated worldwide [2] and to a better overall long-term outcome. The possibility of family planning and having biological children is crucial for long-term survivors, and infertility drastically downgrades quality of life [3].

The risk of infertility due to chemo- or radiotherapy depends on the underlying disease, age, sex, other comorbidities, and the choice and dosage of chemotherapeutics, as well as the determination of the radiation field [4, 5]. For patients undergoing HSCT, gonadotoxicity is mainly associated with alkylating agents (especially busulfan) and total body irradiation (12 Gy) and the overall risk of infertility after HSCT is 70-80% [6]. Female patients who are exposed to gonadotoxic treatment experience subfertility with a diminished ovarian reserve at a younger age than the general female population [6].

### Current practice

The current practice of FP counseling and the performance of FP procedures differ among European countries according to national recommendations, local logistics, technical experience and the assumption of costs by the national insurance system. In France and Israel, FP composes part of national law. In 2012, Nordic countries recommended counseling and the possibility of FP procedures for all patients at risk of fertility injury due to oncology treatment, including HSCT. This program is financed by the national insurance system [7]. In Germany and Switzerland, there has been no assumption of costs by the national insurance system until now. However, both countries recently changed the legal basis for financing FP procedures [8], but especially in Germany, coverage of the costs for patients below 18 years of age is still unclear, although the implementation of FP procedures in this age group was recommended by German AWMF guidelines as early as 2015 [9].

FP counseling is a difficult task for physicians and demands a sensitive approach regarding the ethnic and cultural background of the families as well as the maturity and age of the patient. In particular, adolescents should be addressed directly, as they usually wish to be included in the decision-making process [10]. For children and adolescents, it is important to integrate developmentally appropriate patients in the decision-making process by using tools that enable decision-making and assent in children [11]. In addition to the shock of a newly diagnosed malignant disease or the naming of side effects during a pre-HSCT interview, the risk of infertility can be further devastating news and should be taken into consideration [7]. FP counseling helps in devising a strategy to address the problem in the best way and might provide an option for future fertility.

FP counseling should include:

1. Risk assessment of infertility according to the patient’s age, treatment/conditioning regimen, and known comorbidities with possible impacts on fertility.
2. Presentation of FP techniques eligible for the counselled patient and their established or experimental characteristics.
3. Rational and individualized decision-making with patients/caregivers taking into account the risk of delaying treatment, the risk of transferring malignant cells with gonadal tissue, and the risk of side effects from the FP procedure itself.

### Options for FP in boys and girls

There are some well-established and experimental treatment options available for FP. Cryopreservation of sperm is a standard procedure [12], while cryopreservation of immature testicular tissue remains experimental and should only be performed within study protocols to ensure ongoing research in this field [13]. Recently, the grafting of autologous premature testes of monkeys resulted in adult spermiogenesis and, finally, in the birth of a healthy baby monkey [14]. These results are promising for preserving fertility in prepubertal boys. For postpubertal female patients, the cryopreservation of unfertilized eggs has been an established method of FP for years [15]. If it is not possible to delay the start of therapy for this procedure, cryopreservation of ovarian tissue for later retransplantation is recommended [16]. The American Society for Reproductive Medicine (ASRM) recently concluded that ovarian tissue cryopreservation is an acceptable FP technique and should no longer be considered experimental [17]. In prepubertal female patients, it is the only method for preserving fertility. Some reports have already described children born after retransplantation of cryopreserved prepubertal ovarian tissues [18, 19]. In FP that involves cryopreservation of tissue, the risk of the reintroduction of malignant cells with the cryopreserved tissue has to be discussed with the patients and their families [20]. The risk should be considered low in some cases of solid tumors and Hodgkin lymphomas, intermediate in cases of non-Hodgkin lymphomas and some metastasized solid tumors such as Ewing`s sarcoma or rhabdomyosarcomas. This risk is highest in cases of neuroblastoma and leukemia, primarily acute lymphoblastic leukemia, which was shown in post-mortem tissue analyses as well as in FP cryopreserved ovarian tissue [21, 22].

### Objective

We describe the process of the implementation of fertility counseling and our experience in the Division of Pediatric Oncology and Stem Cell Transplantation using an inhouse standard procedure established by our team. Before we implemented structured fertility counseling in our center, the risk of gonadotoxic side effects was addressed in the initial interview about the disease and the individualized therapy options with the patient and the family. The counseling did not follow a standard format and was not conducted in a separate interview. The aim of this prospective cohort study was to analyze the demographic characteristics of counseled patients, the percentage of FP recommendation and their adoption by specific patients’ cohorts (female vs male, pre- vs post-pubertal, non-malignant vs malignant patient cohort).

Notably, the implementation of a structure for counseling and assessing the performance of FP took place at a time while FP was not yet covered by the German health insurance system. The inability to cover costs and therefore low staffing levels are some reasons why FP counseling has not yet been established in all pediatric units dealing with gonadotoxic treatment.

## Materials and methods

### Patients

This is a prospective cohort study analyzing data collected in a single center with the start of implementation of fertility counseling. From January 2017 to February 2019, we counseled patients with a newly diagnosed malignant disease or who were undergoing HSCT. In our center, we usually see patients 0–18 years old. Nevertheless, we include young adults as part of the CAYA project (care of adolescents and young adults) for oncological treatment. The majority of patients in this study were less than 18 years old, the oldest 22 years at the most.

As part of the initial work-up, all patients underwent a standardized procedure for examination of pubertal status, including clinical examination, abdominal and, if applicable, testicular sonography and hormone measurements (female patients: LH, FSH, estrogen, anti-mullerian hormone; male patients: LH, FSH, testosterone, inhibin B, sex hormone-binding globulin). The Tanner stages, and in boys’ testis volume, were used to determine the clinical stage of puberty. Tanner stages I–II were considered pre-pubertal, Tanner IV–V post-pubertal (including testis volume greater 10ml clinically and by ultrasound). The clinical findings were compared with the results of the hormone levels. Patients with Tanner stage III could be assigned to either the prepubertal or post-pubertal group based on the hormone values. If this was not possible, they were designated as peri-pubertal. All data were collected as part of the initial work-up and documented in the chart. Following informed consent by the caregivers and the patient, the clinical findings, the counseling and the FP recommendation were documented in a database. Prior to implementation of FP counseling, an ethical approval was obtained for this FP database (see below). The data for the present analysis were taken from this database and the medical files.

### In-house standard procedure for fertility counseling and preservation

Before the structured fertility counseling service was established, the team decided to define a uniform recommendation guideline (in-house standard procedure) so that the vast majority of patients would receive counseling based on international recommendations as well as national, local and individual conditions.

With the medical team, we reviewed recent guidelines based on those of the German, Austrian and Swiss Societies of Pediatric Oncology and Hematology [9], the German Society for Gynecology and Obstetrics, the German Society for Urology and German Society for Reproductive Medicine [23], the American Society of Clinical Oncology [24], and the European Society for Blood and Marrow Transplantation [25] in detail. A list of indications for FP procedures was established for all tumor entities and was used as the in-house standard procedure for the counseling process at our institution (Supplementary Table 1). This list defines a treatment-related risk score for fertility impairment for each tumor entity according to the above mentioned guidelines [9, 23–25], and a time window within FP had to be implemented to avoid a delay initiating therapy and the risk of contamination of the tissue due to the primary disease and metastatic status at time of diagnosis.

For each patient, we discussed the risk of the FP procedures regarding the general patient condition.

Especially for very young children with a low gonadotoxic risk (for example hepatoblastoma and some nephroblastoma), we did not recommend experimental techniques. For all systemic malignancies (e.g., leukemias) treated with allo-HSCT, we do not recommend tissue cryopreservation at the moment despite the high gonadotoxic risk of the treatment for the risk of reinducing malignant cells with retransplantation of the tissue [20] (see the “Discussion”). For patients with solid tumors, we recommended considering fertility-preserving methods for all tumors with an intermediate risk of gonadotoxicity (50–70% risk of infertility) [9], such as osteosarcoma and Ewing’s sarcoma, unless there was evidence of multiple metastases. In case of intermediate risk of treatment for non-metastasized tumors, the potential of the FP procedure had to be weighed against the potential risk of damaging gonadal tissue through the procedure itself. Cryopreservation procedures were recommended for all patients with nonmalignant diseases treated with HSCT.

Fertility counseling was offered to the parents of all children and adolescents undergoing chemotherapy or HSCT independent of whether FP was recommended or planned to be performed. One devoted person of the oncology/transplantation team did the counseling, independently from the general informative interview on diagnosis and treatment to ensure the possibility of concentrating solely on the fertility aspect of the diagnosis and treatment plan. The counseling lasted 30–60 min and included a face-to-face interview with the caregivers and the patients. In all patients except for those less 6 years, we aimed to give age at least general information on the fertility aspect of their treatment. With the adolescent patients, we discussed the gonadotoxic risk and FP measures in detail. We also offered written material to further explore the topic (brochures designed by the German Society of Pediatric Oncology and by our Department of Reproductive Medicine).

To perform the FP procedures, we created a multidisciplinary team composed of members of the Division of Pediatric oncology/hematology and stem cell transplantation, Gynecology/Reproductive Medicine, Andrology, Pediatric Surgery and Psychology/ psychosocial team.

In cases of invasive procedures such as ovarian or testicular tissue cryopreservation, we aimed to time these operations with other procedures, such as the implantation of a tunneled central line, because of the risk of side effects owed to anesthesia and the psychological impact of undergoing multiple surgery. In some cases, the invasive fertility-preserving procedures were performed independently from other operations (for example, if the patient already had a central line when being transferred to our department). However, we usually attempted to avoid this due to the experimental nature of the FP procedures for young patients.

### Statistical analysis

Data analysis was performed with R version 3.6.1 (foundation for statistical computing, Vienna, Austria). All tests were two-sided with a significance level of 0.05.

Descriptive statistics for categorical data are presented as absolute frequencies and percentages, and those for continuous data are presented as the median, interquartile range (IQR), maximum and minimum. Fisher’s exact test was used for comparisons between groups with a two-sided Clopper-Pearson 95% confidence interval (CI).

We received approval from the Ethics Committee of the University Hospital Frankfurt/Main (Reg. No. 239/17).

## Results

**Fig. 1:**
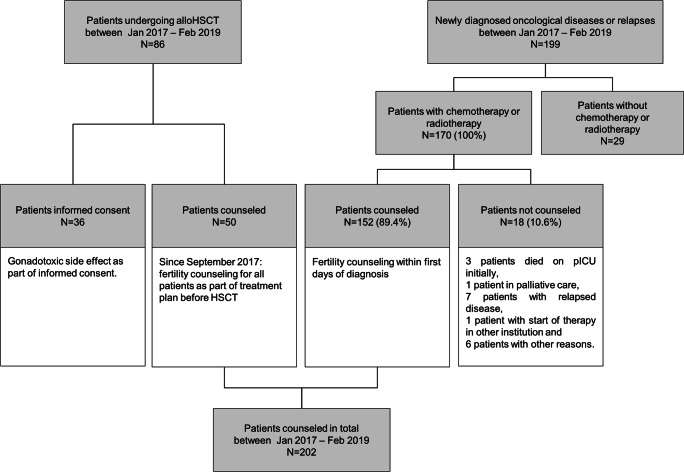
Analysis of patients receiving fertility counseling in our institution between January 2017 and May 2018. Note: Starting in September 2017, patients planned for allo-HSCT received detailed fertility counseling separately from the general informed consent for allo-HSCT

In total, we counseled 202 patients from the Division of Pediatric Hematology and Oncology (152 patients) and the Division of Pediatric Stem Cell Transplantation (50 patients) between January 2017 and February 2019. In this period, 199 new patients were admitted to our oncology department with newly diagnosed oncological diseases (Fig. 1). Of these, 170 were scheduled to receive either chemotherapy, including auto-HSCT, or radiotherapy as part of their treatment plan. A total of 89.4% (*N*= 152) of the 170 oncological patients received fertility counseling within the first days of diagnosis, including detailed information regarding the gonadotoxicity of their treatment, and we offered FP procedures if indicated. In the same manner, we counseled 50 patients who were scheduled to receive allo-HSCT. Usually, the counseling lasted 30–60 min and was performed independently from the counseling regarding diagnosis and treatment planning (Fig. 1).

In the total cohort of 202 counselled patients, 41% (*N* = 82) were girls, and 59% (*N* = 120) were boys. Fifty-nine percent of the counseled girls (*N* = 48) and 69% (*N* = 83) of the boys were prepubertal. Only one female and three male patients could not be determined as being either pre- or postpubertal (Table 1). Table 1 shows the demographic data of the patients regarding sex, age group, disease entities and pubertal status. In our cohort, 59% of the counseled patients were boys; this disparity was especially striking in the leukemia group.

**Table 1:** Patient characteristics and epidemiologic data. Data shown for female and male patients regarding age groups, disease entity, and pubertal status

	**Total**	**Girls**	**Boys**	***P*** **value**
**N (%)**	202 (100)	82 (41)	120 (59)	
FP counselling date				1
Median	Feb 2018	April 2018	Jan 2018	
IQR	July 2017- Sep 2018	June 2017- Oct 2018	July 2017- Aug 2018	
Min - Max	Jan 2017 – Feb 2019	Jan 2017 – Feb 2019	Jan 2017 – Feb 2019	
**Age, years**				0.602
Median	8.5	10	8	
IQR	3 - 14	2 - 14	3 - 14	
Min - Max	0 - 22	0 - 19	0 - 22	
Mean ± SD	8.6 ± 5.6	8.9 ± 5.9	8.9 ± 5.4	
**Age groups, N (%)**				0.070
0 - 3 y	57 (28)	25 (30)	32 (27)	
4 - 6 y	22 (11)	6 (7)	16 (13)	
7 - 10 y	42 (21)	13 (16)	29 (24)	
11 - 13 y	24 (12)	14 (17)	10 (8)	
14 - 16 y	44 (22)	15 (18)	29 (24)	
17 - 22 y	13 (6)	9 (11)	4 (3)	
**Diagnosis, N (%)**				0.052
Leukaemia	69 (34)	18 (22)	51 (43)	
Lymphoma	26 (13)	13 (16)	13 (11)	
Solid tumour	70 (35)	35 (43)	35 (29)	
MDS	5 (3)	1 (1)	4 (3)	
Non-malignancies	28 (14)	13 (16)	15 (13)	
Others	4 (2)	2 (2)	2 (2)	
**Puberty, N (%)**				0.134
Pre-pubertal	132 (65)	48 (59)	84 (70)	
Pubertal	4 (2)	1 (1)	3 (3)	
Post-pubertal	66 (33)	33 (40)	33 (28)	

*FP*, fertility preservation; *IQR* interquartile range; *Min*, minimum; *Max*, maximum; *MDS*, myelodysplastic syndrome

Regarding diagnosis, 35% of the patients presented with malignant solid tumors, while 34% were diagnosed with acute leukemias. Lymphoma was found in 13% of the patients, and myelodysplastic syndrome was found in 3%. Nonmalignant diseases requiring HSCT, such as sickle cell disease, thalassemia major or congenital immunodeficiency syndromes were the underlying medical condition in 14% of patients.

**Fig. 2:**
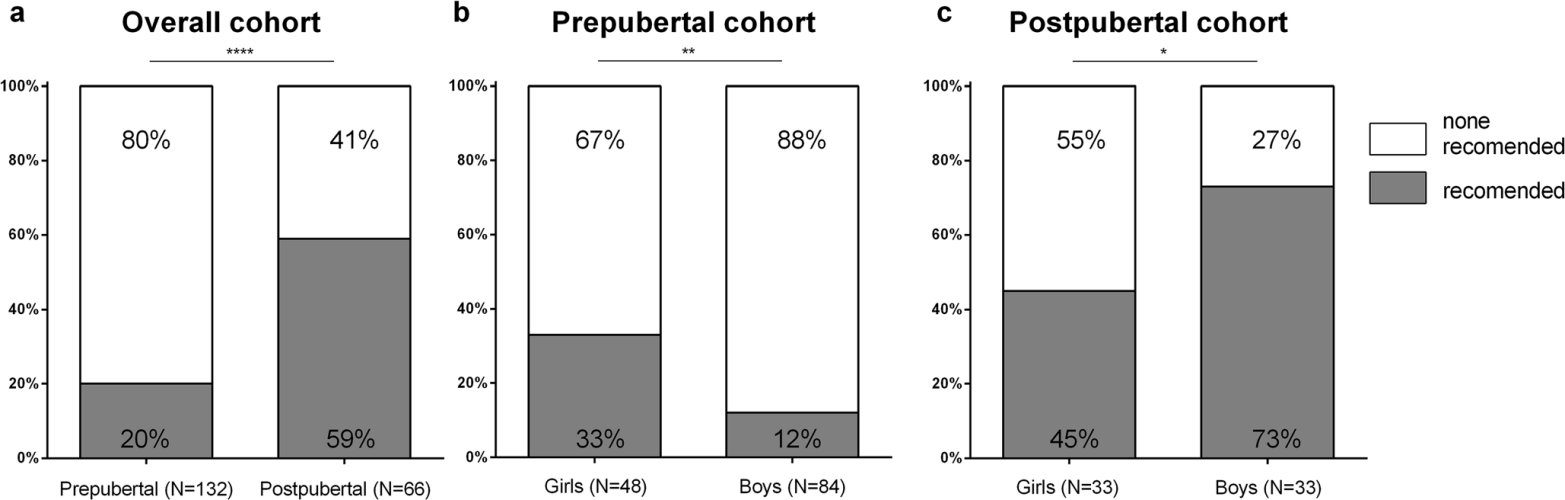
Recommendations of fertility-preserving measures with regard to pubertal status and sex. For better clarity, we excluded peri-pubertal patients (*N*= 1 in girls, *N*= 3 in boys). Significant differences between groups were assessed by Fisher’s exact test. Differences were considered significant for *P* <0.05 (*), *P* <0.01 (**), and *P*<0.001 (***). The light color indicates that no procedure was recommended, and the dark area indicates that a fertility measure was recommended

**Fig. 3:**
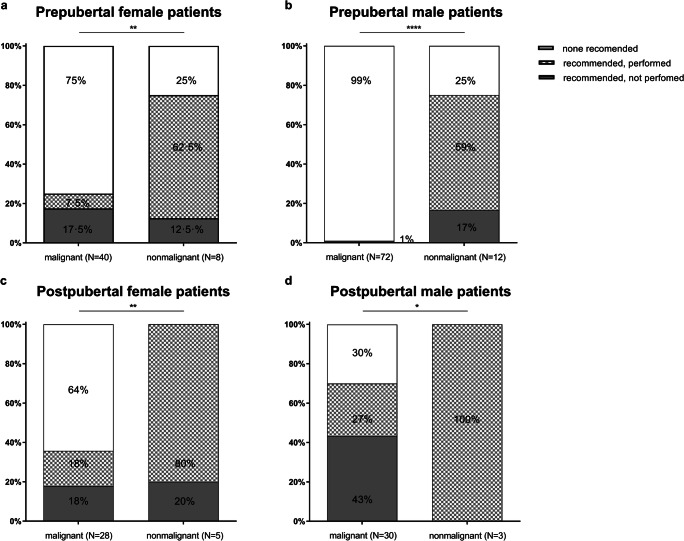
Results of the counseling process: recommended and performed vs. non performed fertility-preserving procedures in prepubertal vs postpubertal female and male patients, separately examining the malignant and non-malignant cohorts. Significant differences between groups were assessed by Fisher’s exact test. Differences were considered significant for *P* <0.05 (*), *P* <0.01 (**), *P*<0.001 (***), and *P*<0.0001 (****). Light color indicates that no measure was recommended, and squares indicate that measures were recommended and performed. Dark color represents a recommended measure that was not performed

The results of the fertility counseling process are shown in Figs. 2 and 3. Figure 2 demonstrates that, as expected, the recommendations for fertility-preserving measures depended on pubertal level and sex. We observed a statistically significant increase in FP recommendations between the age groups from prepubertal (20%; *N*=26; 95% CI 13–28%) to postpubertal status (59%; *N*=39; 95% CI 46–71%) (*P*<0.001) for the whole cohort (Fig. 2A).

Looking at the pubertal subgroups, both the prepubertal and postpubertal groups demonstrated statistically significant differences between the female and male subgroups concerning FP recommendations (Fig. 2B and C). In the prepubertal group, only 33% (*N*=16; 95% CI 20–48%) of the female patients did receive a recommendation for an invasive procedure, and even less, 12% (*N*=10; 95% CI 6–21%) of the prepubertal male patients were counseled likewise (*P*=0.005). In the postpubertal group, this rate of recommendation was 45% (*N*=15; 95% CI 28–64%) vs. 73% (*N*=24; 95% CI 54–87%) (*P*=0.044) for female and male patients, respectively, thus half of the postpubertal female patients and over two thirds of the postpubertal male patients received a recommendation for a FP procedure

In addition, in the female group, there were no statistically significant differences in the number of patients with no recommendation for a FP procedure between the pre- and postpubertal subgroups (67% vs. 55%; *P*=0.069), but in the male group, the FP recommendations depended strongly on the pubertal status (88% vs. 27%; *P*<0.001).

The procedures recommended for postpubertal female patients were almost exclusively ovarian tissue cryopreservation. Only one postpubertal female patient underwent hormonal stimulation for oocyte cryopreservation and succeeded in storing unfertilized oocytes for later in vitro fertilization (see the “Discussion” section).

Figure 3 summarizes the outcome of the counseling process in regard to sex, pubertal stage, and malignant versus nonmalignant disease. In the malignant cohort, we recommended an FP procedure for 25% of the prepubertal female patients, but only 7.5% of the patients and their families agreed to it. In the nonmalignant cohort, we advised 75% of the patients to undergo an invasive procedure according to our in-house standard procedure, and 62.5% agreed to it (Fig. 3A). In the postpubertal female cohort, we recommended an FP procedure to 36% of the malignant cohort, and half of the patients followed our recommendation. In the nonmalignant cohort, we counseled 100% of the patients to undergo a procedure, and 80% followed our recommendation (Fig. 3C). In the male cohort, the difference between pre- and postpubertal patients and between malignant and nonmalignant disease was even more pronounced. In the prepubertal cohort of patients with malignant diseases, we did not recommend a fertility-preserving measure for 99%, while we recommended an invasive procedure for 100% of the nonmalignant cohort, and only 25% of patients did not follow our recommendation (Fig. 3B). We offered sperm cryopreservation to the majority of postpubertal male patients. While 100% of the nonmalignant cohort followed our recommendation, only 27% of the malignant cohort did so, mainly for personal reasons (Fig. 3D). For 9 patients, we did not recommend sperm cryopreservation. Two patients had already started chemotherapy at other institutions, 5 patients had a relapse of their disease (4 leukemia, 1 lymphoma), had already started chemotherapy or received radiation therapy of the testis and had come to our institution for SCT, and two patients were in a poor overall medical condition at the time of counseling. The results of the counseling process for the age subgroups are shown in Supplementary Figure 1 (female patients) and Supplementary Figure 2 (male patients).

No patient in the 11–13 year age group, 52% of the patients in the 14–16 year age group, and 25% of the 17–22 year age group underwent sperm cryopreservation. For one patient, the clinical status worsened; thus, sperm cryopreservation was no longer feasible. In another patient, the organization of cryopreservation would have resulted in a significant delay in the start of treatment. Six patients did not want to undergo this procedure for personal reasons, and for 3 patients, the quality of the sperm was not suitable for cryopreservation. Only one patient underwent testicular biopsy to cryopreserve the sperm.

## Discussion

We present the first data following the initiation and implementation of fertility counseling in our single pediatric hematology/oncology, stem cell transplantation and immunology center in a situation where the costs of FP procedures not yet covered by the national health system in Germany. Before this structured process was started, there was no standardized fertility counseling for patients, and the discussion of the gonadotoxic side effects of cancer treatment formed only part of the general information before seeking informed consent concerning diagnosis and treatment. Despite a broader awareness by the public and the legal chances of financial support for fertility-preserving measures, many pediatric hemato-oncology departments are experiencing a similar situation. After the structuring processes and implementation of a pattern for fertility counseling by the team using an inhouse standard procedure, it was possible to counsel approximately 90% of the newly diagnosed oncological patients and 100% of the patients undergoing HSCT according to our inhouse standard procedure for FP counseling. Patients not counseled included those initially admitted to the Intensive care unit due to a severe medical condition and those from other countries visiting our department only for portions of their treatment. The attending medical team always consulted with the counseling physician if fertility counseling was felt to be of benefit for relapsed patients and their family.

Recommendations regarding FP procedures were well accepted by the patients and their families. For prepubertal patients, especially in the 0–3 year age group, we rarely recommended an invasive procedure. This approach will change in Germany in the coming years. After the implementation of severe combined immunodeficiency (SCID) screening in Germany in 2019, we recorded a number of very young patients (< 1 year) who qualified for HSCT and thus potentially for fertility-preserving measures.

For most patients in the 0–3 year age group with malignant diseases, FP procedure was not recommended due to the low risk of gonadotoxicity of the treatment or a high risk of contamination of the gonadal tissue with malignant cells. The retransplantation of cryopreserved immature gonadal tissue is still experimental, and there is a high risk of contamination with malignant cells in leukemia and certain solid tumors [20]. Further research is needed to determine possibilities in addressing potentially contaminated tissue, such as the in vitro maturation of immature oocytes [26]. Within the multidisciplinary team, we decided at the beginning of our project not to recommend tissue cryopreservation in these cases due to the highly experimental status of the procedure and the fact that health insurance in our country does not cover cryopreservation. Taking these points together, we decided that starting the implementation of the fertility counseling with the highest consensus from the team was important. Within the team, we agreed on the processes for reevaluating this decision and the consequences of our in-house standard procedure on a regular basis with the opportunity to change indications for invasive procedures in case of new research data and/or changes in coverage of the costs by the national health insurance.

Nevertheless, gonadal tissue collection from patients with leukemia can be considered, in view of future developments, for in vitro maturation and subsequent in vitro fertilization, and we offered it to families at their request. Notably, however, none decided to undergo the procedure.

In the subgroup of very young children, included patients with abdominal tumors (hepatoblastomas, neuroblastomas, nephroblastomas), abdominal surgery for ovarian tissue harvesting was thought to add potential complications. In some cases, the children were already critically ill; thus, we agreed with the parents not to place the child at further risk with an invasive procedure.

For prepubertal male patients, there is only the possibility of cryopreserving immature testicular tissue. Due to the experimental nature of the procedure and the risk for malignant cell infiltration, we recommend it only in cases with a high risk of gonadotoxicity for nonmalignant diseases (see the discussion above concerning contamination of gonadal tissue with malignant cells), almost exclusively for patients facing HSCT. The tissue was sent to the tissue bank of the University of Muenster as part of the FP project Androprotect [27]. In these cases, the percentage of patients who accepted undergoing a recommended measure was remarkably high (75%) despite its experimental nature. Reasons not to perform a recommended and patient-approved procedure in older patients were associated either with worsening of the patient’s medical condition or personal reasons of the patients. Only in rare cases were logistical reasons involved, e.g., timing of invasive procedures without a delay in initiating chemotherapy. Strikingly, the acceptance for sperm cryopreservation was 100% in the nonmalignant teenage cohort, while that in the malignant group was quite low. The reasons and implications of this difference are currently under investigation in our institution. Though the numbers of our cohort are still small (e.g., *N*=3 in the non-malignant male subgroup), this experience is consistent with other studies investigating the use of sperm cryopreservation in young adults [28, 29]. However, the majority of these patients in this study chose not to bank sperm or were not offered the opportunity. The authors discus a range of factors such as time, emotional state, patient age, disease stage, institutional practices, and cost all influence whether banking is offered to patients and taken up. Although the possibility for FP exists in our center, a detailed discussion took place and the costs of cryopreservation were covered for a certain period of time, only a few patients took advantage of the possibility. We speculate as well that this was due to the eminent irritation and distress of a newly diagnosed oncological disease. In these cases, we need to find ways to help teenaged male patients understand the importance of cryopreserving material and thus undergo sperm cryopreservation. Biopsy of the post-pubertal testis is a feasible method if it is not possible to produce sperm spontaneously. For patients who did not succeed in producing sperm in sufficient number or quality, we discussed this method with the patient/family. In most cases, we agreed not to use this procedure due to its invasive nature (in addition to the need for anesthesia) and the possible delay of treatment by planning this procedure. It would be interesting to determine if more young men with malignant disease would agree to cryopreserve their sperm as part of a more distant procedure with less personal involvement.

Cryopreservation of oocytes in female patients is an established method of FP [30]. However, in our cohort, only one patient underwent this procedure. Cryopreservation of unfertilized oocytes requires 12–14 days of hormonal stimulation. Our oncological team agreed on a tight time frame for FP procedures, with a maximum delay of 7 days for the start of chemotherapy. There is no clear evidence regarding the degree to which different delays negatively influence the outcome [31]; thus, decisions such as these needs to be reevaluated on a regular basis. For patients with nonmalignant diseases such as thalassemia major, oocyte cryopreservation is a feasible method [32]. Patients with sickle cell disease are at risk of a sickle cell crisis triggered by hormonal stimulation; therefore, oocyte cryopreservation is generally not recommended for these patients [33].

### Implications of findings

An in-house standard procedure for fertility-preserving procedures eased the implementation of fertility counseling at our institution and provided consistent yet individualized counseling for patients facing gonadotoxic treatment for either malignant or nonmalignant disease.

Other institutions have been able to show that after the structured establishment of fertility counseling, significantly more patients underwent FP procedures [34]. However, in a recent survey of FP counseling, even in EBMT settings for pediatric HSCT with a high risk of gonadotoxicity, less than half of the patients received FP counseling, and less than one third underwent FP procedures [35]. This demonstrates the need for broad FP programs. Our intention in establishing an in-house standard procedure was to enable comprehensive consultation for all pediatric patients with gonadotoxic risk in our pediatric clinic. In contrast to departments that care for adult patients facing gonadotoxic therapy, efforts to provide comprehensive counseling for children and their families remain in the early stages in many countries [7]. In-house standard procedures with indication lists for the fertility counseling of young patients based on a common consensus of the caring team and on recent guidelines would, in our view, simplify implementation considerably and simultaneously create awareness of the FP possibilities prior to gonadotoxic therapy. Meanwhile, an in-house standard procedure would help in determining which pediatric patients FP measures are indicated without delaying the start of therapy or imposing an additional risk through invasive measures for the child.

The implementation of fertility counseling using an inhouse indication list as a standard procedure could potentially be adapted first in national and later international settings to allow a more standardized handling of the counseling while allowing a consensus regarding the counseling within the multiprofessional team to be formed. However, this study describes the results of a single center experience, and adaptation through other institutions is pending to show that it is feasible to implement counseling for FP with an in-house standard operating procedure (SOP) according to recent FP guidelines. Ongoing evaluation of the counseling process should constantly improve it in terms of the needs of the patients and families.

In times of limited medical staff and time resources, it is important to ensure adequate scheduling for fertility counseling within the first days of oncological diagnosis. Therefore, additional funding sources are needed. In our case, counseling was enabled through the funding of a parents’ association. In our institution, we have a multidisciplinary team composed of members of all departments involved in FP. There is effective cooperation with the departments of gynecology/reproductive medicine, andrology, pediatric surgery, and psychology. Within this network, it is possible to ensure quick and individual discussions of newly diagnosed patients and the realization of FP procedures if indicated. A multidisciplinary team that involves strong coordination and collaboration in the abovementioned fields is essential to guarantee FP for pediatric patients [36].

Official guidelines and recommendations (AWMF, ASCO, EBMT, and ARSM) represent the current medical opinion regarding FP. As they rely on former and recent data, it is important to assure ongoing discussion of recent research results in a young field such as FP for gonadotoxic therapy. Furthermore, ethical questions regarding how to counsel patients with genetic disorders as well as patients with disabilities are emerging issues that require discussion.

## Supplementary information

Suppl. Table 1 Inhouse SOP for fertility preserving in regard to disease, metastasis, time frame, specific fertility preserving measures for female and male patients. Note, the risk assessment of the gonadotoxic risk indicated in dark grey (high risk), light grey (intermediate risk), stripes (intermediate-low) and white (low). +: procedure recommended, (+): procedure suitable to only a limited extent, –: procedure not recommended, **#:** scientific data controversial, *: indication only if radiation therapy of abdomen/gonads/spine, ^**1** :^ performance within study protocol of approved study, experimental in prepubertal children, not indicated if patients are in bad condition or at higher risk for intraoperative complications. Palliative care: individual approach after consulting palliative care team, parents, patient (DOCX 218 kb)

Suppl. FIGURE 1 Data analysis of the counseled female patients. Number of counseled patients, number of procedures recommended, and number of procedures performed. Data are shown for the entire group of patients and by age group. (A) Prepubertal female patients. (B) Postpubertal female patients. All procedures shown consisted of ovarian tissue biopsy and either pre- or postpubertal cryopreservation. For better clarity we excluded peri-pubertal patients (N= 1). Not included: the sole female patient who underwent oocyte cryopreservation (see text). (PNG 290 kb)

High resolution image (TIF 1852 kb)

Suppl. FIGURE 2 Data analysis of the counseled male patients. Number of counseled patients, number of procedures recommended, and number of procedures performed. Data are shown for the entire group of patients and by age group. (A) Prepubertal male patients. All performed procedures consisted of biopsy and cryopreservation of immature testes according to the study protocol. (B) Postpubertal male patients. All performed procedures consisted of sperm cryopreservation, and no testicular biopsies were performed. For better clarity we excluded peri-pubertal patients (N= 3). (PNG 310 kb)

High resolution image (TIF 1879 kb)

## Data Availability

All data related to this study are available on request.

## Abbreviations

ASCO: American Society of Clinical Oncology
ASRM: American Society for Reproductive Medicine
AWMF: Arbeitsgemeinschaft der Wissenschaftlichen Medizinischen Fachgesellschaften e.V.
EBMT: European Society for Blood and Marrow Transplantation
