# Supplementary material for: How can fertility counseling be implemented for every newly diagnosed pediatric patient facing gonadotoxic treatment?-A single-center experience
Source: Ann Hematol. 2021 Sep 18;100(11):2831–41. doi: 10.1007/s00277-021-04648-z (PMC8510917; doi:10.1007/s00277-021-04648-z)
Supplement: Supplementary file 1 — Inhouse SOP for fertility preserving in regard to disease, metastasis, time frame, specific fertility preserving measures for female and male patients. Note, the risk assessment of the gonadotoxic risk indicated in dark grey (high risk), light grey (intermediate risk), stripes (intermediate-low) and white (low). +: procedure recommended, (+): procedure suitable to only a limited extent, –: procedure not recommended, #: scientific data controversial, *: indication only if radiation therapy of abdomen/gonads/spine, 1 : performance within study protocol of approved study, experimental in prepubertal children, not indicated if patients are in bad condition or at higher risk for intraoperative complications. Palliative care: individual approach after consulting palliative care team, parents, patient (DOCX 218 kb) [file 277_2021_4648_MOESM1_ESM.docx]

Suppl. Table 1

|  |  | **MALE** | | | **FEMALE** | | |  |
| --- | --- | --- | --- | --- | --- | --- | --- | --- |
|  |  | Postpubertal | Pubertal / Postpubertal | Prepubertal | Postpubertal | Postpubertal (after menarche) | Prepubertal / Postpubertal | Prepubertal / Postpubertal |
| Disease | Maximum time of treatment delay | Sperm cryopreservation | TESE | Biopsy immature testis^1^ | Oocyte cryopreservation | GnRH agonist# | ovarian tissue cryopreservation^1^ | Ovariopexy* |
| SCT malignant disease | depending on disease | + | (+) | – | (+) | (+) | – | – |
| SCT non-malignant disease | mostly flexible | + | + | + | + | (+) | + | – |
| Radiation of pelvis and spine in solid tumors | 1 week | + | + | + | (+) | (+) | + | + |
| Osteosarcoma | 1 week | + | + | + | (+) | (+) | + | + |
| Ewing sarcoma | 1 week | + | + | + | (+) | (+) | + | + |
| + multiple metastasis |  |  |  | – |  |  | – |  |
| Hodgkin's disease | One week, stadium III/IV only if clinical good condition | + | + | – | (+) | (+) | + | + |
| Nephroblastoma | 1 week | + | + | – | (+) | (+) | (+) | + |
| Soft tissue-sarcoma | 1 week | + | (+) | (+) | (+) | (+) | (+) | + |
| + multiple metastasis |  |  |  | – |  |  | – |  |
| Neuroblastoma | 1 week | + | (+) | (+) | (+) | (+) | (+) | + |
| + multiple metastasis |  |  |  | – |  |  | – |  |
| Brain tumor + chemotherapy | 1 week | + | (+) | (+) | (+) | (+) | (+) | + |
| Retinoblastoma | 1 week | + | – | – | (+) | (+) | – | – |
| Acute leukemia, Non-Hodgkin-Lymphoma | 1 day | + | – | – | – | (+) | – | – |
| Germ cell tumor (ovary/testis) | 5 days | + | – | – | – | (+) | – | + |
| High grad glioma | 1 week | + | See palliative care | See palliative care | See palliative care | (+) | See palliative care | – |
| Hepatoblastoma | 1 week | + | – | – | (+) | (+) | – | – |
| Langerhans Cell Histiocytosis | 1 week | + | (+) | – | (+) | (+) | – | – |

| **+** **procedure recommended** |
| --- |
| **(+) procedure suitable to only a limited extent** |
| **– procedure not recommended** |

| **Risk stratification according to recent guidelines** |
| --- |
| red field: high gonadotoxic risk of therapy (>70%) |
| yellow field: intermediate gonadotoxic risk of therapy (50-70%) |
| green-yellow field: intermediate-low gonadotoxic risk of therapy   (depending on disease and risk stratification) |
| green field: low gonadotoxic risk of therapy (<20%) |

Abbrev: SCT, stem cell transplantation; TESE, testicular sperm extraction.

**#:**  scientific data controversial

***:** indication only if radiation therapy of abdomen/gonads/spine

**^1^**  ^:^ performance within study protocol of approved study. Experimental in prepubertal children. Not indicated if patients are in bad condition or at higher risk for intraoperative complications

**Palliative care**: individual approach after consulting palliative care team, parents, patient

**Suppl. FIGURE 1**. Data analysis of the counseled female patients. Number of counseled patients, number of procedures recommended, and number of procedures performed. Data are shown for the group of patients in total and in the distribution of age groups. (A) Prepubertal female patients. (B) Postpubertal female patients. All procedures shown are ovarian tissue biopsy and cryopreservation either prepubertal or postpubertal. Not included is the one female patient who underwent oocyte cryopreservation (see text).

**Suppl. FIGURE 2**. Data analysis of the counseled male patients. Number of counseled patients, number of procedures recommended, and number of procedures performed. Data are shown for the group of patients in total and in distribution of age groups. (A) Prepubertal male patients. All performed procedures consisted of biopsy and cryopreservation of immature testis according to study protocol. (B) Postpubertal male patients. All performed procedures consisted of sperm cryopreservation, no testicular biopsies were performed.
