# Supplementary figures and images for: How can fertility counseling be implemented for every newly diagnosed pediatric patient facing gonadotoxic treatment?-A single-center experience
Source: Ann Hematol. 2021 Sep 18;100(11):2831–41. doi: 10.1007/s00277-021-04648-z (PMC8510917; doi:10.1007/s00277-021-04648-z)

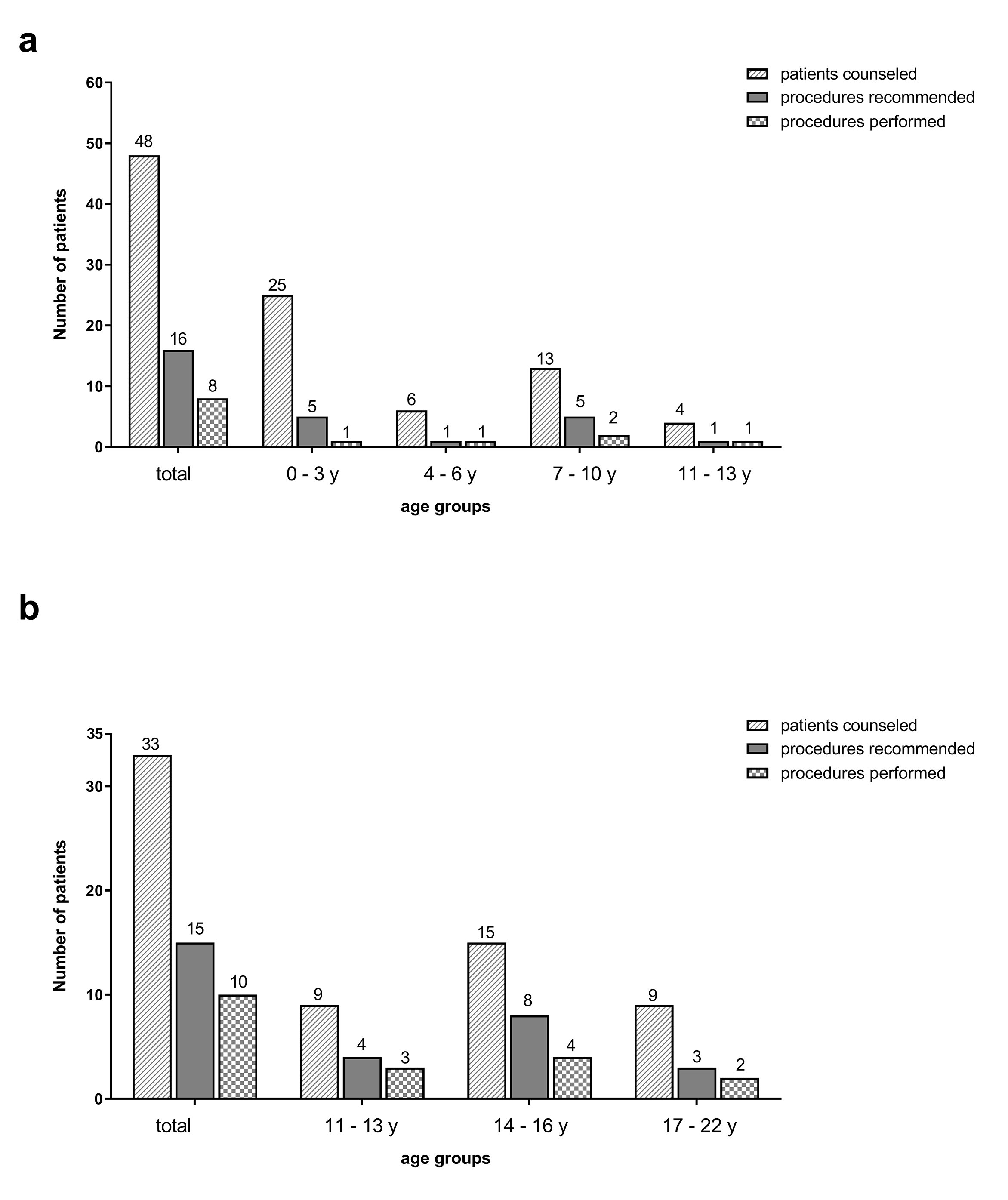

Supplement: Supplementary file 2 — Data analysis of the counseled female patients. Number of counseled patients, number of procedures recommended, and number of procedures performed. Data are shown for the entire group of patients and by age group. (A) Prepubertal female patients. (B) Postpubertal female patients. All procedures shown consisted of ovarian tissue biopsy and either pre- or postpubertal cryopreservation. For better clarity we excluded peri-pubertal patients (N= 1). Not included: the sole female patient who underwent oocyte cryopreservation (see text). (PNG 290 kb) [file 277_2021_4648_Fig4_ESM.png]

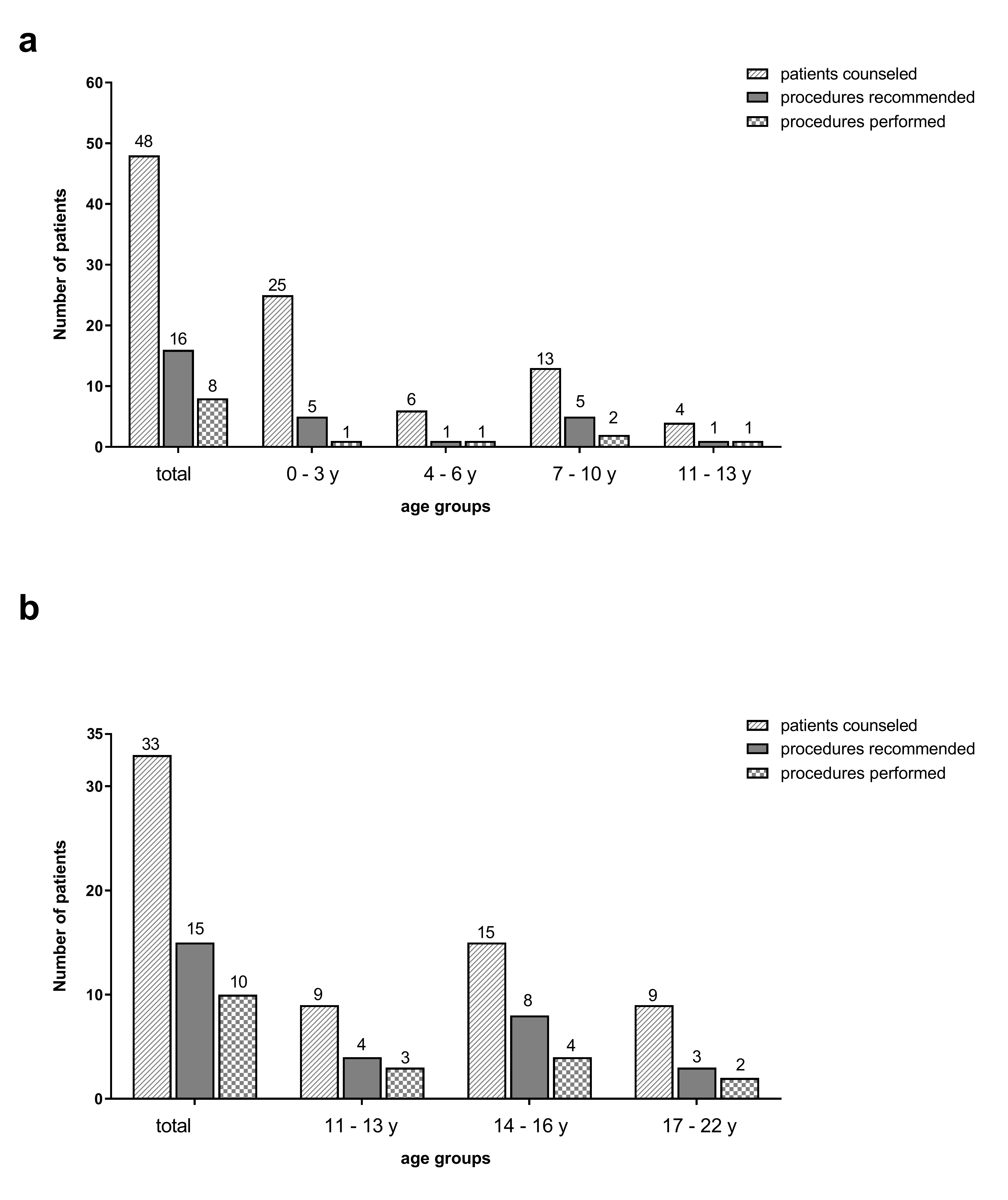

Supplement: Supplementary file 3 — High resolution image (TIF 1852 kb) [file 277_2021_4648_MOESM2_ESM.tif]

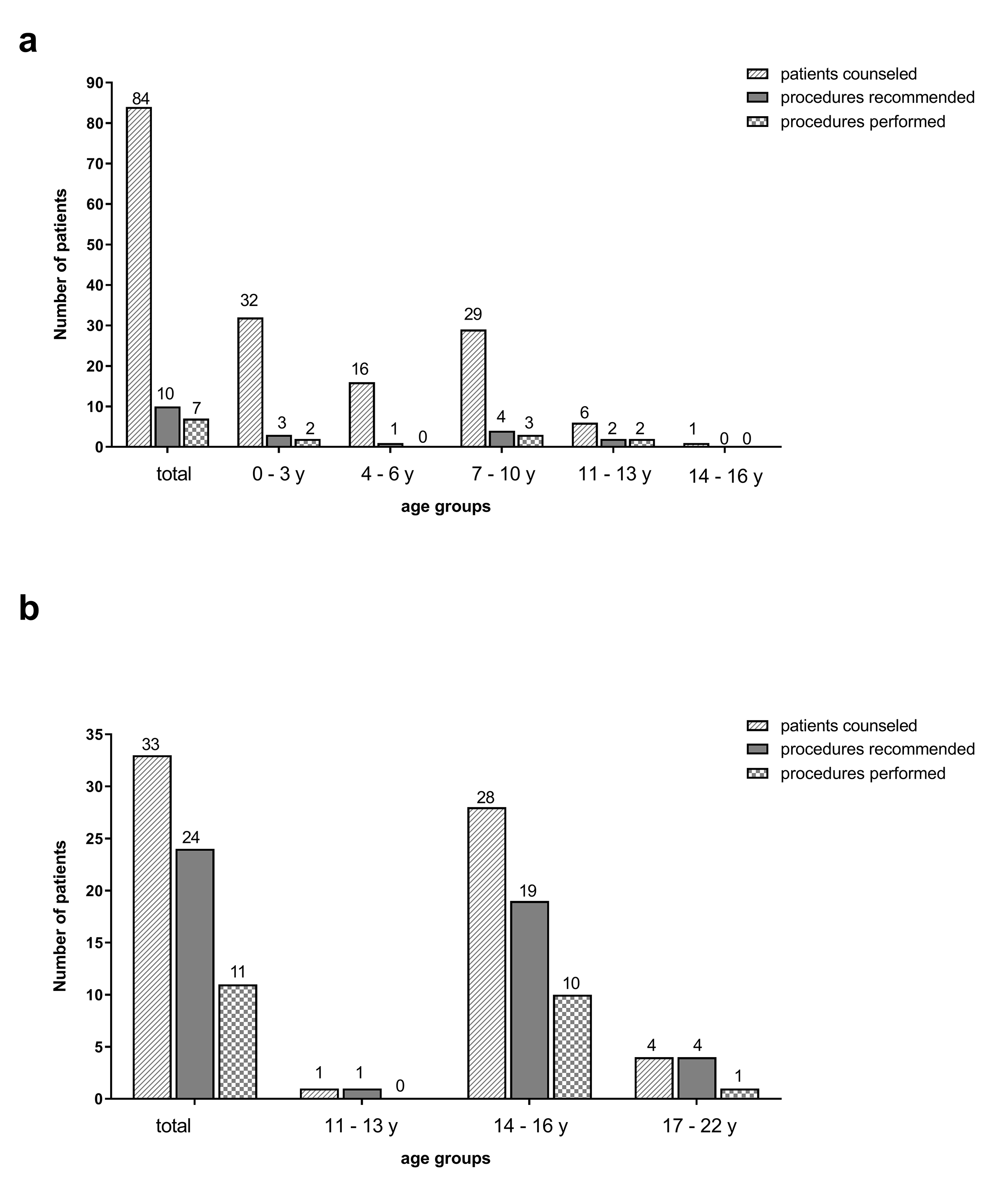

Supplement: Supplementary file 4 — Data analysis of the counseled male patients. Number of counseled patients, number of procedures recommended, and number of procedures performed. Data are shown for the entire group of patients and by age group. (A) Prepubertal male patients. All performed procedures consisted of biopsy and cryopreservation of immature testes according to the study protocol. (B) Postpubertal male patients. All performed procedures consisted of sperm cryopreservation, and no testicular biopsies were performed. For better clarity we excluded peri-pubertal patients (N= 3). (PNG 310 kb) [file 277_2021_4648_Fig5_ESM.png]

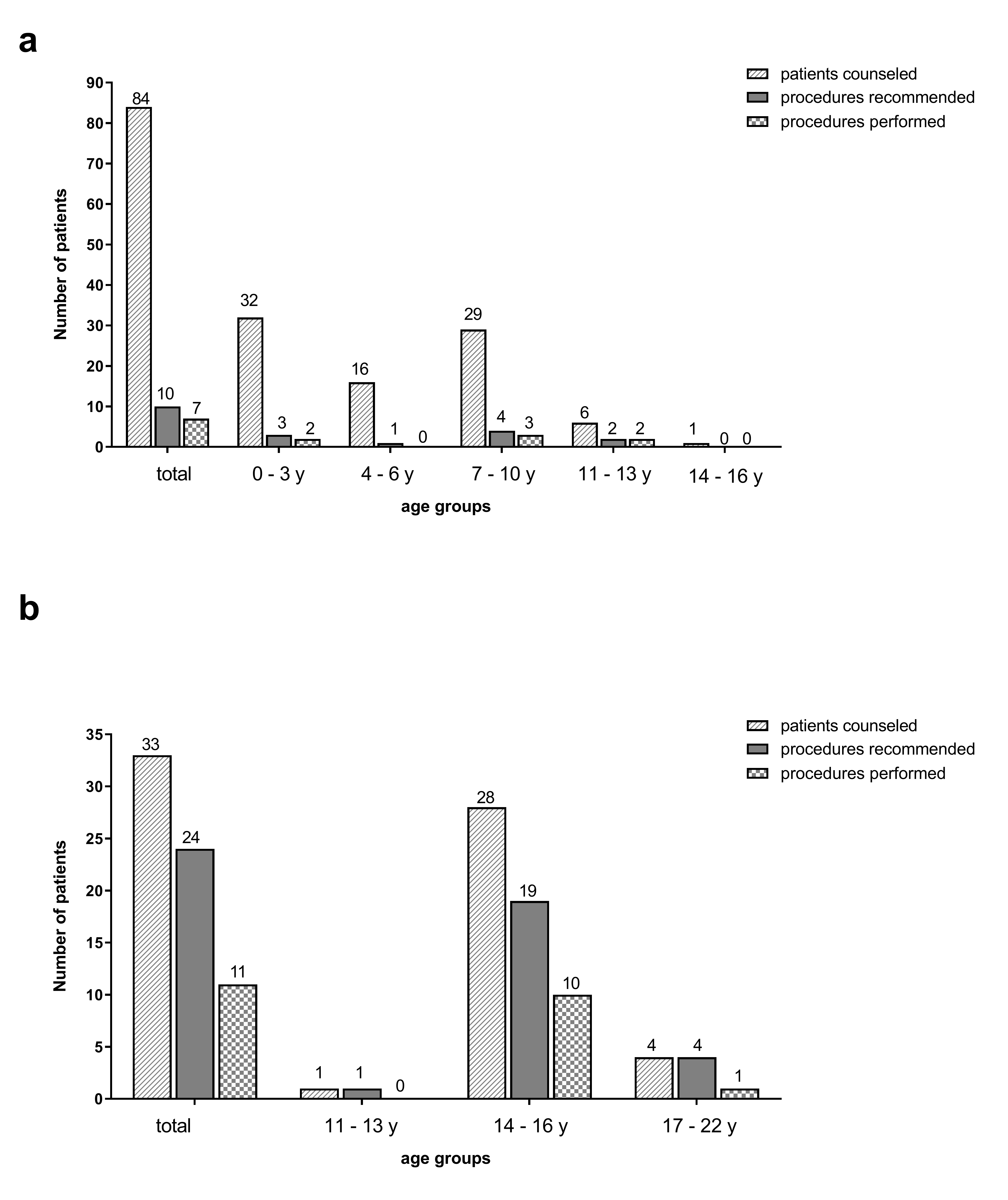

Supplement: Supplementary file 5 — High resolution image (TIF 1879 kb) [file 277_2021_4648_MOESM3_ESM.tif]
